# Inhibitory immune checkpoints suppress the surveillance of senescent cells promoting their accumulation with aging and in age-related diseases

**DOI:** 10.1007/s10522-024-10114-w

**Published:** 2024-07-01

**Authors:** Antero Salminen

**Affiliations:** Department of Neurology, Institute of Clinical Medicine, University of Eastern Finland, P.O. Box 1627, 70211 Kuopio, Finland

**Keywords:** Ageing, Cellular senescence, Immunosenescence, Immunosuppression, Immunosurveillance, Inflammaging

## Abstract

The accumulation of pro-inflammatory senescent cells within tissues is a common hallmark of the aging process and many age-related diseases. This modification has been called the senescence-associated secretory phenotype (SASP) and observed in cultured cells and in cells isolated from aged tissues. Currently, there is a debate whether the accumulation of senescent cells within tissues should be attributed to increased generation of senescent cells or to a defect in their elimination from aging tissues. Emerging studies have revealed that senescent cells display an increased expression of several inhibitory immune checkpoint ligands, especially those of the programmed cell death protein-1 (PD-1) ligand-1 (PD-L1) proteins. It is known that the PD-L1 ligands, especially those of cancer cells, target the PD-1 receptor of cytotoxic CD8^+^ T and natural killer (NK) cells disturbing their functions, e.g., evoking a decline in their cytotoxic activity and promoting their exhaustion and even apoptosis. An increase in the level of the PD-L1 protein in senescent cells was able to suppress their immune surveillance and inhibit their elimination by cytotoxic CD8^+^ T and NK cells. Senescent cells are known to express ligands for several inhibitory immune checkpoint receptors, i.e., PD-1, LILRB4, NKG2A, TIM-3, and SIRPα receptors. Here, I will briefly describe those pathways and examine whether these inhibitory checkpoints could be involved in the immune evasion of senescent cells with aging and age-related diseases. It seems plausible that an enhanced inhibitory checkpoint signaling can prevent the elimination of senescent cells from tissues and thus promote the aging process.

## Introduction

A chronic low-grade inflammation and immunosenescence are common immune-related hallmarks of the aging process; this remodelling phenomenon has been called inflammaging (Franceschi et al. 2018). While many age-related and chronic inflammatory diseases display similar properties, it does seem that these immune alterations are clearly augmented in these diseases. Nonetheless, the cellular state of senescence is a common feature encountered in both the aging process and many age-related diseases (Yanai and Fraifeld 2018; Mylonas and O'Loghlen 2022). Senescent cells are pro-inflammatory cells which secrete a number of inflammatory mediators, such as cytokines, chemokines, complements, and colony-stimulating factors (Rodier et al. 2009; Freund et al. 2010; Hernandez-Segura et al. 2018; Sikora et al. 2021). The pro-inflammatory phenotype has been called the senescence-associated secretory phenotype (SASP) (Campisi and Robert 2014). Currently, it seems that there are multiple causes to explain why cells will switch on the senescence phenotype which displays diverse pro-inflammatory properties in a context-dependent manner (Sikora et al. 2021; Wang et al. 2024a). For instance, the inducer of the senescent state and the cell type involved have a crucial role in the heterogeneity of the SASP (Wang et al. 2024a). Recently, several single-cell transcriptome studies have confirmed that many cell types isolated from aged tissues display a pro-inflammatory phenotype thus verifying results observed in cultured cells (Salzer et al. 2018; Benayoun et al. 2019; Zou et al. 2021; Xu et al. 2022). Moreover, the senescence in aged tissues has revealed the presence of a robust heterogeneity within cell populations. The single-cell transcriptomic studies also confirmed that an increased expression of senescence markers in isolated cells was associated with an enhanced expression of SASP factors. These studies verified that senescent cells are an important source of the elevated expression of pro-inflammatory factors in aged tissues.

The chronic inflammatory microenvironment, either associated with aging or as encountered in many inflammatory diseases, stimulates a counteracting immunosuppression in an attempt to maintain tissue homeostasis and thus avoid tissue destruction (Kanterman et al. 2012; Hotchkiss et al. 2013; Wang and DuBois 2015; Salminen 2020). There is abundant evidence that with aging the number of immunosuppressive cells was significantly increased in tissues. For instance, several investigators have revealed that the populations of regulatory T cells (Treg), myeloid-derived suppressor cells (MDSC), and M2 macrophages were augmented in aged tissues (Grizzle et al. 2007; Enioutina et al. 2011; Jackaman et al. 2013; Garg et al. 2014; Ruhland et al. 2016). The counteracting immune responses also involve the activation of inhibitory immune checkpoint pathways in many pathological states. The host tissues can inhibit the cytotoxic effects of CD8^+^ T and natural killer (NK) cells by increasing the expression of inhibitory checkpoint ligands which suppress the activity of these cytotoxic cells (Buckle and Guillerey 2021; Baldanzi 2022). Currently it is known that there are several inhibitory checkpoint receptor/ligand systems possessing different properties and distributions in many immune cells. The blockade therapies for certain inhibitory checkpoints are clinically used in cancer immunotherapy (Guo et al. 2023). Emerging studies have revealed that senescent cells can evade immune surveillance by activating the expression of the ligands for the programmed cell death protein 1 (PD-1) receptor, i.e., the PD-L1 protein on their cell surface (Onorati et al. 2022; Wang et al. 2022a; Salminen 2024). However, there are several other inhibitory checkpoint receptor/ligand systems which are associated with the immune surveillance of senescent cells. Here, I will briefly introduce those pathways which are known to control the immunosurveillance of senescent cells and examine whether they could be involved in the immune evasion of senescent cells associated with aging.

## Accumulation of senescent cells increases with aging and in age-related diseases

Since the characterization of the major properties of senescent cells in cultured cells, there has been mounting interest to determine whether the same molecular markers could be upregulated in aging tissues. There is growing evidence indicating that the aging process is associated with a progressive increase in the expression of common markers of cellular senescence not only in the tissues of mammalian species (Krishnamurthy et al. 2004; Wang et al. 2009; Biran et al. 2017; Yanai and Fraifeld 2018; Idda et al. 2020; Yousefzadeh et al. 2020) but also in zebrafish, *Caenorhabditis elegans*, and *Drosophila* (Kishi 2004; Dmitrieva and Burg 2007; Ito and Igaki 2016). Moreover, single-cell transcriptome techniques have been used to confirm the presence of age-related senescence phenotypes in the cells of many murine tissues (Tabula Muris Consortium 2020; Zou et al. 2021). Several new techniques have recently become available to help to reveal in vivo the senescence process. For axample, there are now many reporter models that can monitor the gradual increase of senescence in aging mice (Gorgoulis et al. 2019; Gonzalez-Gualda et al. 2021). It seems that an age-related increase in senescence is a tissue-specific process which is greatly augmented in the mouse models of accelerated aging (Yousefzadeh et al. 2020; Cai et al. 2022). Interestingly, several mouse models of an accelerated aging have demonstrated that the removal of senescent cells from tissues actually attenuate pathological alterations taking place in aging tissues (Baker et al. 2016; Yi et al. 2023). For instance, Baker et al. (2011) developed a transgenic mouse model with an inducible elimination of the p16INK4a-positive cells, a common senescence marker (INK-ATTAC model). They demonstrated that both in wild-type and in the accelerated aging background (the BubR1 progeroid mouse) the depletion of senescent cells attenuated and delayed the appearance of many age-related pathologal changes. Currently there are many drug discovery programs aiming to develop pharmacological medications to eliminate senescent cells, i.e., senolytic therapies (Chaib et al. 2022; Zhang et al. 2023).

Many age-related diseases are associated with an expansion of cellular senescence although it seems that cellular senescence does not play the same role in different diseases. For example, it is known that cellular senescence can possess both tumor-suppressive and tumor-promoting properties in a phase-dependent manner (Sun et al. 2018; Takasugi et al. 2022). Given that senescent cells have pro-inflammatory properties, there is mounting evidence that cellular senescence acts as a driver of several age-related diseases, such as many cardiac diseases (Mehdizadeh et al. 2022), skin pathologies (Chin et al. 2023), atherosclerosis (Wu et al. 2020), and hepatic steatosis (Ogrodnik et al. 2017). The accumulation of fibrotic lesions is a significant pathological characteristic in certain age-related diseases. For example, there is clear evidence that cellular senescence, especially the senescence of stromal fibroblasts, enhanced the pathogenesis of fibrotic diseases, e.g., the clearance of senescent fibroblasts reduced the severity of fibrotic pulmonary lesions in mice (Schafer et al. 2017). Cellular senescence also has a crucial role in chronic kidney disease (Xu et al. 2020), myocardial fibrosis (Osorio et al. 2023), and systemic sclerosis (Tsou et al. 2022). It seems that cellular senescence may have a double-edge role in fibrotic diseases, i.e., it can either prevent or promote fibrosis, a feature also observed in tumor biology. Krizhanovsky et al. (2008) demonstrated that in murine hepatic liver fibrosis the presence of senescent hepatic stellate fibroblasts limited the progression of fibrosis and even facilitated the reversal of this pathological trait.

Currently, it is not known why senescent cells accumulate within tissues during aging and age-related diseases. Eventually, it could be attributable to an increase in the generation of senescent cells or to their reduced removal via impaired immune surveillance. There are diverse experimental insults which can switch cells to adopt an irreversible state of senescence. Interestingly, it is known that senescent cells can induce bystander senescence in the neighboring cells within tissues (Nelson et al. 2012; da Silva et al. 2019). This paracrine senescence is most likely induced by the secretion of SASP factors, such as ROS and cytokines. However, senescent cells possess an increased capacity to secrete extracellular vesicles (EV) which not only target neighboring cells but via the systemic circulation they can expand throughout the whole organism (Nelson et al. 2018; Alibhai et al. 2020; Salminen et al. 2020). Krtolica et al. (2001) demonstrated that senescent fibroblasts promoted the growth of epithelial cells and accordinly stimulated tumorigenesis in mice. Interestingly, there are observations indicating that the EVs secreted from senescent cells are able to enhance immunosuppression in chronic inflammatory conditions (Salminen et al. 2020). It is known that EVs can carry a diverse cargo, e.g., many immunosuppressive miRNAs and other suppressive enhancers, such as ARG1, PGE2, and TGF-β proteins. In fact, the EVs released by tumor cells contain membrane-bound PD-L1 proteins which can act as decoys to confuse the anti-PD-L1 antibodies and thus induce a resistance to immunotherapy (Yin et al. 2021; Chen et al. 2022).

There has been an active debate on the role of the immune system in the elimination of senescent cells, i.e., whether immune cells enhance or prevent the removal of senescent cells. Currently, it is known that there are differences in the clearance process of senescent cells between the microenvironments, such as aging tissues, tumors, and fibrotic lesions (Wang et al. 2022a; Chen et al. 2023; Marin et al. 2023; Lee et al. 2024) (see below). There are several reports indicating that impairments in the function of cytotoxic immune cells, i.e., CD8^+^ T and NK cells, promote to the accumulation of senescent cells within aged tissues. For instance, Ovadya et al. (2018) demonstrated that deficiency in immunosurveillance activity, evoked either via genetic or pharmacological treatments, disturbed the clearance of senescent cells (SA-β-gal and p16-positive) from tissues in aged mice. The knockout of the perforin gene (*Prf*^*−*/−^), a cytolytic protein expressed in cytotoxic CD8^+^ T and NK cells, (i) increased cellular senescence in mouse liver, lung, and skin, (ii) enhanced chronic inflammation, and (iii) promoted age-related disorders in several mouse tissues. Moreover, the lack of the *Prf* gene in progeroid mice shortened their lifespan. They also confirmed that the cytotoxicity of CD8^+^ T cells was robustly declined in these transgenic mice. These observations indicate that functional deficiencies in surveying immune cells promote the accumulation of senescent cells within aged tissues.

The age-related immunosenescence has been associated with a robust decline in the cytotoxic activity of CD8^+^ T and NK cells that might impair the elimination of senescent cells (Salminen 2021; Brauning et al. 2022). There is clear evidence that the aging process is associated with activation of the immunosuppressive network intended to inhibit the functions of cytotoxic immune cells (Ruhland et al. 2016; Salminen 2020). Currently, it is known that the age-related immunosuppression can be associated with (i) an increase in the recruitment and differentiation of immunosuppressive cells, such as Tregs, MDSCs, and M2 macrophages, as well as (ii) the activation of inhibitory immune checkpoint signaling, e.g., in the accumulating senescent cells (see below). It is recognized that the immunosuppressive cells secrete anti-inflammatory cytokines, such as TGF-β and IL-10, which inhibit the surveillance activity of CD8^+^ T and NK cells (Li et al. 2006; Ouyang et al. 2011). For instance, Trzonkowski et al. (2006) demonstrated that Tregs inhibited the cytotoxic activity of human CD8^+^ T and NK cells. Accordingly, Filippi et al. (2008) revealed that TGF-β exposure suppressed the activation of mouse naïve CD8^+^ T cells but not that of antigen experienced T cells. Moreover, TGF-β exposure inhibits the cytotoxic activity of NK cells in many experimental models (Viel et al. 2016; Slattery et al. 2021). Nunez et al. (2018) reported that human M2 macrophages reduced the cytotoxic activity of NK cells through the secretion of TGF-β. In their seminal study, Ruhland et al. (2016) demonstrated that senescent stromal cells in mouse skin enhanced inflammation and recruited immunosuppressive Tregs and myeloid-derived suppressor cells (MDSC) into mouse skin. They revealed that senescent stroma in mouse skin robustly decreased immune surveillance of cancer cells and consequently the immunosuppressive environment increased tumorigenesis. Recently, it has been demonstrated that not only immunosuppressive cells but also inhibitory immune checkpoints, such as the PD-1/PD-L1 signaling pathway, can suppress the immune surveillance of senescent cells and thus promote their accumulation with aging (Wang et al. 2022a).

## Inhibitory immune checkpoint signaling suppresses immunosurveillance

The immune system drives immune surveillance in the host intended to recognize foreign pathogens as well as aberrant cells, such as malignant and senescent cells. Cytotoxic CD8^+^ T cells, NK cells and macrophages are the major surveying immune cells which can detect and eliminate not only invading microbes but also abnormal host cells. Antigen presentation via the major histocompatibility complexes (MHC) and the activation of cytotoxic cells are highly regulated processes which are controlled by multilevel regulatory systems (Paul and Lal 2017; Shah et al. 2021). The immune checkpoint receptors fine-tune this process by either stimulating or inhibiting the function of cytotoxic cells. In fact, many checkpoint receptors function as co-receptors which cooperate to maintain the immune balance by regulating the activity of major signaling pathways of cytotoxic cells, such as T cell receptor (TCR) signaling (Baldanzi 2022). For instance, the inhibitory immune checkpoint receptors have an important role in the suppression of autoimmunity disorders, whereas an excessive activity of inhibitory checkpoints prevents the clearance of tumor cells (Toor et al. 2020; Burke et al. 2023).

### Inhibitory immune checkpoint receptors

**Fig. 1:**
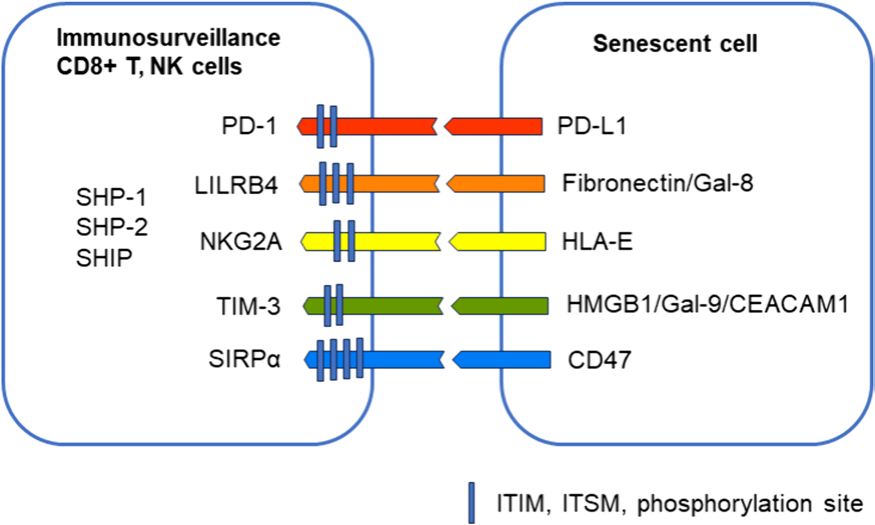
A schematic presentation of the inhibitory immune checkpoint receptors in cytotoxic immune cells and their ligands in senescent cells. *CEACAM 1* carcinoembryonic antigen-related cell adhesion molecule 1, *Gal* galectin, *HLA-E* human leukocyte antigen-E, *HMGB1* high mobility group box 1, *ITIM* immunoreceptor tyrosine-based inhibitory motif, *ITSM* immunoreceptor tyrosine-based switch motif, *LILRB4* leukocyte immunoglobulin-like receptor B4, *NKG2A* natural killer cell receptor G2A, *PD-1* programmed cell death protein-1, *PD-L1* programmed death-ligand 1, *SIRPα* signal regulatory protein α, *TIM-3* T cell immunoglobulin and mucin domain-3

The receptors of the inhibitory immune checkpoints are ligand-activated receptors which are expressed mainly in cytotoxic T and NK cells and macrophages (Ravetch and Lanier 2000; Buckle and Guillerey 2021; Baldanzi 2022; Guo et al. 2023). There are a number of different receptors which possess inhibitory immune activity but I will focus only on those five receptors which are known to act as inhibitory immune checkpoint receptors for the ligand proteins expressed in senescent cells, i.e., programmed cell death protein-1 (PD-1), leukocyte immunoglobulin-like receptor B4 (LILRB4), natural killer cell receptor G2A (NKG2A), T cell immunoglobulin and mucin domain-3 (TIM-3), and signal regulatory protein α (SIRPα). The inhibitory checkpoint receptors are membrane-bound glycoproteins which contain an extracellular Ig-like V domain and a cytoplasmic tail which encompasses the immunoreceptor tyrosine-based inhibitory motifs (ITIM) and tyrosine-based switch motifs (ITSM) (Xu et al. 2021; Baldanzi 2022; Xiang et al. 2024) (Fig. 1). The binding of the ligand to the inhibitory checkpoint receptor induces the phosphorylation of the ITIM or ITSM sites, thus providing the docking sites for the Src-homology-2 (SH2) protein phosphatases, e.g., SHP-1, SHP-2, and SHIP (Xu et al. 2021; Baldanzi 2022; Xiang et al. 2024). Subsequently, these activated phosphatases can dephosphorylate the components of the TCR and costimulatory CD28 pathways, thus suppressing signaling through the TCR axis (Mizuno et al. 2019; Baldanzi 2022). The inhibition of TCR signaling suppresses the activation, proliferation, and cytokine production of CD8^+^ T cells, thus attenuating immune surveillance and cytotoxic activity. Intriguingly, similar defects in the properties of CD8^+^ T and NK cells are typical changes encountered in the immunosenescence state which is not only associated with the aging process but also found in tumors and age-related diseases (Barbe-Tuana et al. 2020; Lian et al. 2020; Salminen 2021). Currently, it seems probable that an increase in the expression of ligands for inhibitory checkpoint receptors (discussed below) could reduce the functional efficiency of the immune system and promote immunosenescence. Next, I will briefly describe the properties of certain inhibitory checkpoint pathways and examine whether an increase in the expression of their ligands in senescent cells could impair immunosurveillance and consequently promote the accumulation of senescent cells within aged tissues.

### PD-1

The PD-1 receptor (CD279), a member of the immunoglobulin superfamily, is expressed on activated T and B cells, NK cells, and some myeloid cells (Ghosh et al. 2021; Beenen et al. 2022). The two ligands for the PD-1 receptor, PD-L1 (CD274 or B7-H1) and PD-L2 (CD273 or B7-DC), are members of the B7 family of immune regulatory ligand proteins (Collins et al. 2005). The PD-L1 proteins are expressed in several antigen presenting and immunosuppressive cells as well as in many stromal cells, e.g., fibroblasts, pericytes, epithelial, endothelial, and smooth muscle cells (Ghosh et al. 2021, Human Protein Atlas). Moreover, the PD-L1 protein is expressed in senescent cells (Onorati et al. 2022; Wang et al. 2022a) and especially in diverse cancer cells and cancer-associated fibroblasts (CAF) (Jiang et al. 2019; Pei et al. 2023). In tumors, the PD-1/PD-L1 checkpoint signaling has a crucial role in the immune evasion of tumor cells and in the generation of an immunosuppressive microenvironment. The binding of the PD-L1 protein to the PD-1 receptor of CD8^+^ T cells activates an inhibitory signaling cascade which leads to a loss of cytotoxicity and even promotes the exhaustion of CD8^+^ T cells. The PD-1/PD-L1 checkpoint axis possesses both beneficial and harmful properties, e.g., it confers immunosuppressive protection in autoimmune diseases, whereas in cancers and fibrotic diseases it provides an immune escape mechanism for abnormal cells to avoid immunosurveillance (Francisco et al. 2010; Toor et al. 2020; Zhao et al. 2023). Currently, the inhibition of the PD-1/PD-L1 signaling with PD-1 and PD-L1 antibodies is a widely used anti-cancer therapy (Tang et al. 2022). However, therapy with PD-1/PD-L1 blockers can induce an immune-based resistance which is due to a loss of PD-L1 expression and defects in antigen presentation and interferon signaling (Vesely et al. 2022). It is known that many combination therapies can improve the treatment response to PD-1/PD-L1 checkpoint inhibitors.

### LILRB4

The LILRB4 receptor (ILT3, gp49B) belongs to the leukocyte immunoglobulin-like receptor subfamily B and it contains three inhibitory ITIM domains (Redondo-Garcia et al. 2023; Xiang et al. 2024). The LILRB4 receptor is expressed in T cells, neutrophils, monocytes, macrophages, dendritic cells, microglia, and endothelial cells. The five members of the LILRB family have different specificities for their activating ligands, i.e., the LILRB4 receptor is activated by fibronectin, apolipoprotein E (ApoE), and galectin-8 (Gal-8) (Redondo-Garcia et al. 2023). The activation of LILRB4 signaling enhances the development of an immunosuppressive microenvironment in mouse and human tumors and it facilitates the immune escape of tumor cells (Sharma et al. 2021). Wang et al. (2024b) demonstrated that an exposure to Gal-8 induced the differentiation of mouse monocytes into monocytic MDSCs and subsequently enhanced their expansion through the activation of STAT3 and inhibition of NF-κB signaling. They also revealed that the activation of LILRB4 promoted tumor growth in mice, whereas antibodies blocking the interaction between Gal-8 and LILRB4 receptor suppressed tumor growth in mice. Accordingly, Deng et al. (2018) reported that the signaling via the ApoE/LILRB4/SHP-2/Arginase-1 pathway in mouse myeloid leukemia cells suppressed T cell activity and created an immunosuppressive microenvironment conducive to tumor invasion. Given that the activation of LILRB4 induces immune tolerance, it seems that LILRB4 signaling may well exert beneficial effects in autoimmune diseases, transplantation biology, and maternal–fetal interactions (Xiang et al. 2024).

Fibronectin, a protein abundantly present in the extracellular matrix (ECM), is also a physiological ligand for the immunosuppressive LILRB4 receptor (Itoi et al. 2022; Itagaki et al. 2023; Xiang et al. 2024). In their seminal study, Itoi et al. (2022) demonstrated that fibronectin was tethered with integrins and the LILRB4 receptor on the cell surface of human and mouse macrophages. The terminal 30 kD domain of fibronectin (FN30) interacted with the LILRB4 receptor, whereas the RGD motif could bind the β-integrin protein. Subsequently, they revealed that the cytotoxicity of mouse NK cells was attenuated when the LILRB4 receptor was activated (Itagaki et al. 2023). These experiments indicated that FN30/LILRB4 signaling inhibited the integrin-assisted natural cytotoxicity of NK cells. In addition to some other inhibitory immune checkpoints in NK cells, such as NKG2A (see below), it seems that FN30/LILRB4 signaling can mitigate the cytotoxicity of NK cells and thus inhibit the elimination of cancer cells. Moreover, Paavola et al. (2021) demonstrated that the fibronectin/LILRB4 interaction inhibited the antigen-presentation by human dendritic cells as well as suppressing cytokine production by the Fcγ receptor (FcR)-activated human THP-1 macrophages. These inhibitory effects could be reversed by treatment with an antibody to the LILRB4 receptor. Paavola et al. (2021) also reported that the blockade of LILRB4 signaling robustly increased the secretion of chemokines, such as CCL3, CCL5, and CXCL10, from tumor cells. There is mounting evidence that fibronectin-activated LILRB4 signaling has profound effects on immune responses therefore LILRB4 signaling is a promising target in drug discovery.

### NKG2A

The NKG2A receptor is a member of the C-type lectin-like receptor superfamily which includes seven NKG2 receptors, both inhibitory and activating checkpoint receptors (Guo et al. 2023). For instance, the NKG2A receptor is an inhibitory checkpoint in immunosurveillance, whereas the NKG2D receptor is a potent activator of the immune clearance of cancer and senescent cells (Iannello and Raulet 2013; Sagiv et al. 2016). The NKG2A receptor is abundantly expressed on the surface of human NK and CD8^+^ T cells. Currently, the HLA class I histocompatibility antigen, α chain E (HLA-E) is the sole ligand for the NKG2A receptor (Lee et al. 1998). The expression of the HLA-E protein is enriched on the surfaces of unhealthy cells, such as infected cells as well as tumor and senescent cells. Ljubic et al. (2023) examined the molecular mechanisms in the binding process between the HLA-E ligand and the NKG2A receptor and they revealed that the inhibitory signaling pathways of the NKG2A receptor were mediated via its two ITIM domains. There is clear evidence that the NKG2A/HLA-E complex inhibits the immune surveillance and impairs the clearance of tumor cells by the cytotoxic NK and CD8^+^ T cells (Andre et al. 2018; Kamiya et al. 2019; Fisher et al. 2022). The blockade of the interaction between the HLA-E and the NKG2A receptor is an important drug discovery target since it can unleash the anti-tumor immunity of cytotoxic NK and CD8^+^ T cells (Andre et al. 2018; Kamiya et al. 2019). It has been claimed that the immunosuppressive NKG2A/HLA-E axis has an important role in many immune-mediated diseases, such as in tumors, infections, and autoimmune diseases (Wang et al. 2022b).

### TIM-3

The TIM-3 receptor, also called hepatitis A virus cellular receptor 2 (HAVCR2), is expressed in T, NK, NKT, and many myeloid cells (Wolf et al. 2020; Buckle and Guillerey 2021). There are several ligands for the TIM-3 receptor, i.e., galectin-9 (Gal-9), phosphatidylserine (PtdSer), high mobility group protein B1 (HMGB1), and carcinoembryonic antigen cell adhesion molecule-1 (CECAM-1) (Wolf et al. 2020; Buckle and Guillerey 2021). The Gal-9 protein is also a ligand for another inhibitory immune checkpoint receptor, i.e., the V-domain immunoglobulin suppressor of T cell activation (VISTA) (Shekari et al. 2023). While the cytoplasmic tail of the TIM-3 receptor lacks the ITIM or ITSM domains, it does contain five conserved tyrosine residues which mediate the downstream inhibitory signaling. The function of TIM-3 is associated with (i) dysfunction and exhaustion of CD8^+^ T cells by disturbing TCR signaling, (ii) inhibition of the activity of Th1 and Tc1 cells, (iii) induction of antigen-specific tolerance, (iv) suppression of some properties of NK cells, (v) increase in transplantation tolerance, and (vi) inhibition of many activities of the innate immune system (Anderson et al. 2016; Wolf et al. 2020; Buckle and Guillerey 2021). In autoimmune diseases, IFN-β therapy increased the expression of TIM-3 and thus enhanced protective immunity, whereas in chronic viral infections and cancers, TIM-3 expression repressed immunosurveillance and inhibited the immune clearance (Anderson et al. 2016; Wolf et al. 2020). Currently, the TIM-3 receptor is a promising target for immunotherapy, especially in many tumors (Sauer et al. 2023).

The Gal-9 ligand of the TIM-3 receptor is a secreted, β-galactoside-binding protein which also has important intracellular functions linked with its lectin property, e.g., Gal-9 can enhance cytosolic ubiquitination and thus activate AMP kinase (Jia et al. 2020; Wolf et al. 2020). As a ligand for the TIM-3 receptor, Gal-9 was shown to promote immunosuppression, e.g., in human cancers, by triggering an exhaustion of CD8^+^ T cells and impairing the cytotoxicity of NK cells (Kandel et al. 2021; Yang et al. 2021). Moreover, the Gal-9 protein can enhance the differentiation of immunosuppressive Tregs, whereas it reduces the level of Th17 cells and thus attenuates the severity of mouse collagen-induced arthritis (Seki et al. 2008). The HMGB1 ligand of the TIM-3 receptor is a multifunctional alarmin localized in chromatin structures which is secreted in many pathological states (Tang et al. 2023). When it gains access to the extracellular space, HMGB1 can interact with many immunoreceptors, both immunosuppressive and immunostimulatory. For instance, HMGB1-mediated TIM-3 signaling was able to inhibit the innate immunity responses to nucleic acids induced by Toll-like receptors 3, 7, and 9 in mouse tumors (Chiba et al. 2012). Moreover, it is known that the HMGB1 protein is an important mediator of the senescence phenotype (Davalos et al. 2013). DeKruyff et al. (2010) demonstrated in mice that PtdSer, a membrane phospholipid and a marker of apoptotic cells, is a ligand for the TIM-3 receptor. There are studies indicating that the TIM-3 receptor mediates the phagocytosis of apoptotic cells and consequently induces the cross-presentation of antigens from dying cells (Nakayama et al. 2009; DeKruyff et al. 2010). The membrane-bound CEACAM1 protein is the fourth ligand for the TIM-3 receptor expressed in many immune and non-immune cells (Wolf et al. 2020). CEACAM1 is a multifunctional glycoprotein which has diverse other functions independent of TIM-3 signaling, e.g., it can enhance cell–cell adhesion and control insulin clearance (Kuespert et al. 2006). CEACAM1 also regulates several TIM-3-dependent immune functions. For example, Zhang et al. (2017) demonstrated that CEACAM1 and TIM-3 promoted the exhaustion of CD8^+^ T cells in colorectal cancer patients. Moreover, the expression of the CEACAM1 protein in activated mouse NK cells inhibited the cytolytic activity of the NKG2D receptor (Hosomi et al. 2013). It seems that it is difficult to develop immunotherapy based on CEACAM1 protein because it is involved in many crucial immune and metabolic functions.

### SIRPα

The SIRPα protein is a membrane-bound inhibitory checkpoint receptor which is mainly expressed in myeloid cells, especially in macrophages, although it can also be present in stem cells and neurons (Barclay and Van den Berg 2014; Logtenberg et al. 2020). The SIRPα receptor interacts with the CD47 protein, also called the integrin associated protein (IAP), which is widely expressed in both immune and non-immune cells (Barclay and Van den Berg 2014; Isenberg and Montero 2024). The major function of the SIRPα/CD47 axis is to inhibit the phagocytosis of apoptotic and abnormal cells, i.e., the CD47 protein transmits a “don’t eat me” signal to macrophages via the activation of SIRPα. The cytoplasmic tail of SIRPα contains four conserved tyrosine residues which act as an ITIM domain to block the phagocytosis of apoptotic cells by macrophages (Logtenberg et al. 2020). Tumor cells as well as senescent cells utilize this mechanism to avoid their phagocytosis by macrophages (Jia et al. 2021; Schloesser et al. 2023). The CD47 protein is a potent marker of the “self” and for instance, the Cd47-induced Sirpα signaling in mouse NK cells prevented immunosurveillance and the NK cell-mediated destruction of xenogeneic tissues (Deuse et al. 2021). Moreover, it is known that cancer cells can pack the CD47 protein into extracellular vesicles and thus evade immune surveillance (Shimizu et al. 2021). The activation of SIRPα is reputed to be involved in the pathogenesis of many diseases, such as atherosclerosis, chronic viral infections, and tumor growth (Feng et al. 2023). In addition, the CD47 protein can also serve as a receptor for the matricellular thrombospondin 1 (TSP1) protein which can trigger the CD47-mediated downstream responses thus inducing disturbances in gene expression, mitochondrial function, and many metabolic activities (Roberts and Isenberg 2021). Currently, the downstream signaling mechanisms of the CD47 protein still need to be clarified.

### Other inhibitory checkpoint receptors

There are many other inhibitory checkpoint receptors in the immune system (Baldanzi 2022; Guo et al. 2023) although currently there is not enough relevant evidence on whether their ligands are expressed in senescent cells. For instance, many human Siglec receptors contain tyrosine motifs and the ITIM domain and they display many of the properties associated with inhibitory immune checkpoint receptors, e.g., Siglec 5, 7, 10, 15 (Ravetch and Lanier 2000; Guo et al. 2023). It is known that the Siglec/sialic acid axis acts as a glyco-immune checkpoint which can inhibit the functions of many surveying immune cells (Saini et al. 2022) and it has a crucial role in many human diseases (Duan and Paulson 2020). For instance, it is known that cancer cells exploit sialoglycan-Siglec interactions to evade immunosurveillance (van de Wall et al. 2020). However, currently it is not known whether cellular senescence affects the surface sialylation of senescent cells in a way which could provide immune evasion for senescent cells. However, several investigators have reported that the level of many sialoglycans decreases rather than increases with cellular senescence (Tadokoro et al. 2006; Itakura et al. 2016).

## Senescent cells evade immune clearance by increasing the expression of ligands for inhibitory immune checkpoint receptors

As described above, the ligands of checkpoint receptors have a crucial role in the immune prevention of host tissues. It seems that cancer cells and senescent cells are able to evade immune surveillance by increasing their expression of the ligand proteins for inhibitory checkpoint receptors. It is known that signaling of many inhibitory checkpoint receptors suppresses the activation of the TCR or B cell receptors (BCR) (Okazaki et al. 2001; Mizuno et al. 2019). There are studies indicating that senescent cells can also increase their antigen presentation by stimulating the expression of proteins of the MHC-I/II machinery (van Tuyn et al. 2017; Chen et al. 2023; Marin et al. 2023; Sinning et al. 2023). For instance, Marin et al. (2023) demonstrated that the cellular senescence induced by three different senescence inducers (genotoxic, CDK4/6 inhibitor, and p53 activator) robustly upregulated antigen presentation via the MHC-I machinery in human IMR-90 fibroblasts and mouse embryonic fibroblasts (MEF) as well as in two human melanoma cell lines. Moreover, tumor senescent cells induced the production of immunogenic peptides which were able to activate CD8^+^ T-dependent antitumor immunity. However, the immune surveillance performed by CD8^+^ T and NK cells is dependent on the balance between the function of the stimulatory and inhibitory co-receptors in these cytotoxic cells and it is this balance that determines whether a senescent cell will or will not be eliminated (Chen and Flies 2013; Zhang and Vignali 2016; Baldanzi 2022).

Interestingly, Chen et al. (2023) reported that the p53 activation-induced senescence of mouse hepatic tumors and tumor cells removed their immune resistance and increased their immune clearance. They also revealed that IFNγ signaling promoted the immune surveillance and elimination of senescent tumor cells. Bojko et al. (2019) demonstrated that different chemotherapeutic agents induced diverse senescence phenotypes in respect to different drugs and cancer cell types. It seems that the inducer of senescence state has a crucial role in tumor cell immunogenicity. For instance, Lee et al. (2024) demonstrated that the senescence of human breast cancer cells induced by CDK4/6 inhibitors promoted immunogenic anti-tumor properties, whereas treatment with DNA-damaging agents augmented the pro-tumorigenic microenvironment. This difference was attributable to an increase in the level of SASP factors produced by DNA-damaging treatment. This is an important observation since it is known that pro-inflammatory SASP factors are present in aged tissues and they activate the expression of many inhibitory immune checkpoints, e.g., that of PD-L1 in aged mice, and thus increase the ability of senescent cells to avoid surveillance by cytotoxic immune cells (Wang et al. 2022a). Next, I will examine the expression of the ligands of certain inhibitory immune checkpoint receptors in senescent cells and aged tissues and their effects on the immunosurveillance of senescent cells.

### PD-1/PD-L1

There is clear evidence that the expression levels of PD-L1 are increased with aging in many mouse and human tissues, such as cerebellum, cardiac muscle, kidney, liver, lung, and spleen (Benayoun et al. 2019; Garcia et al. 2022; Onorati et al. 2022; Wang et al. 2022a). This is not surprising since it is known that pro-inflammatory SASP mediators are potent inducers of the expression of PD-L1 protein (Antonangeli et al. 2020; Betzler et al. 2020; Rong et al. 2021; Nawas et al. 2023; Salminen 2024). It has been reported that the NF-κB system, especially the non-canonical RelB signaling, as well as JAK-STAT signaling stimulates the expression of the PD-L1 protein (Antonangeli et al. 2020; Onorati et al. 2022; Zhang et al. 2022). Moreover, the NLRP3 inflammasomes, when primed by NF-κB signaling, were observed to be involved in the upregulation of PD-L1 expression and immunosuppression in mouse lymphoma (Lu et al. 2021). Given that the SASP-related inflammatory markers are augmented in many age-related diseases, there are reports indicating that the expression of PD-L1 is upregulated in coronary artery disease (CAD), obstructive lung disease (COPD), and Alzheimer’s disease (AD) (Saresella et al. 2012; Weyand et al. 2018; Polverino et al. 2022). Moreover, it is known that the expression of PD-L1 has an important role in the development of age-related fibrotic lesions in cardiac muscle, kidney, and lungs (Jiang et al. 2022; Onorati et al. 2022; Zhao et al. 2023).

The accumulation of senescent cells seems to be one source of the increased expression of the PD-L1 protein in aged tissues. Onorati et al. (2022) demonstrated that the exposure of cultured human IMR-90 fibroblasts to a wide variety of different inducers of cellular senescence, e.g., replicative exhaustion, etoposide, oncogenic HrasV12, and ionizing radiation, stimulated a robust increase in the expression of the PD-L1 protein in senescent fibroblasts. A cytochemical evaluation of the fibroblasts revealed that there was an intense staining of the PD-L1 protein at the cell-surface of senescent fibroblasts. While it is known that the PD-L1 protein can also accumulate into the nuclei (Hudson et al. 2020), these results clearly indicated that after senescence treatments, the PD-L1 protein was localized to the cell-surface and thus was able to target the PD-1 receptor of immune cells. Interestingly, Onorati et al. (2022) reported that the SASP factors released from cultured senescent fibroblasts were able to induce an upregulation of PD-L1 expression in neighboring cells. They also revealed that secreted factors induced the expression of PD-L1 via the JAK-STAT pathway. Using different imaging techniques, Wang et al. (2022a) demonstrated that with aging the PD-L1-positive cells accumulated within mouse liver and kidney. They also observed that the expression level of PD-L1 correlated with the degree of SASP markers in cells of aged tissues. Interestingly, Wang et al. (2022a) demonstrated that those cells with a high expression level of the PD-L1 protein in cultured mouse pulmonary fibroblasts were protected against the immune surveillance and clearance by cytotoxic CD8^+^ T cells. This observation indicates that an increase in the expression of the PD-L1 protein in senescent cells was able to reduce the elimination of senescent cells by cytotoxic immune cells.

### LILRB4/Fibronectin/Gal-8

Fibronectin is an abundant glycoprotein which binds not only to ECM proteins, such as collagens and some proteoglycans, but also to the cell-membrane bound LILRB4 and integrin receptors (see above). There are many investigations revealing that the aging process affects the properties of fibronectin in aged tissues, i.e., (i) its expression level increases in several cell types in aged tissues (Boyer et al. 1991; Kumazaki et al. 1993; Wicher et al. 2021), (ii) the expression of fibronectin is activated by NF-κB-mediated inflammatory signaling (Qwarnström et al. 1994; Lee et al. 2002), and (iii) the age-related structural alterations occurring in the fibronectin matrix increase the rigidity of the aged ECM (Antia et al. 2008). Interestingly, already two decades before the characterization of the SASP, an increased expression and secretion of fibronectin was identified as a marker of cellular senescence (Shevitz et al. 1986; Smith and Pereira-Smith 1989; Kumazaki et al. 1991). Kumazaki et al. (1993) reported that human fibroblasts in the skin and in human vascular endothelial cells displayed a similar upregulation in the expression of fibronectin both in aged tissues and in cell cultures of replicative senescence. Accordingly, Wang et al. (2015) demonstrated that the replicative senescence of mouse cardiac fibroblasts displayed comparable fibrogenic changes, i.e., an increase in the expression of fibronectin and collagen 1A1 and 3A1, as were evident in the fibroblasts present in the fibrotic myocardium of aged mice. The deposition of fibronectin into tissues in conjunction with pro-inflammatory senescent cells might have a protective role in many inflammatory diseases, e.g., atherosclerosis and autoimmune diseases, since the activation of LILRB4 signaling inhibits NF-κB signaling and moreover, it promotes the activation of immunosuppressive MDSCs (Zhou et al. 2020; Singh et al. 2021; Liu et al. 2023). The pathway of LILRB4, but not those of the integrins, was most probably involved since treatment with the antagonist of the LILRB4 receptor reversed the suppression of T cells induced by human monocytic MDSCs (Singh et al. 2021). Zhou et al. (2020) demonstrated that the activation of LILRB4 signaling protected mouse cardiac muscle from fibrosis and hypertrophy through an inhibition of NF-κB signaling.

Galectins (Gal) are a family of the β-galactoside-binding lectins which have important functions in both intracellular and extracellular spaces. For instance, the Gal-8 protein inhibits the activity of mammalian target of rapamycin (mTOR), an essential regulator of metabolism and physiology (Jia et al. 2018). The Gal-8 protein is highly expressed and secreted in inflammatory conditions and it can regulate many innate and adaptive responses (Tribulatti et al. 2020). Wang et al. (2024b) demonstrated that the Gal-8 protein is a functional ligand for the LILRB4 receptor and it is able to induce the differentiation of M-MDSCs from mouse monocytes by activating the STAT3 pathway and inhibiting NF-κB signaling. The Gal-8 protein is robustly expressed in cancer cells and it is a significant enhancer of the LILRB4-mediated immunosuppressive milieu and the growth of tumors (Wang et al. 2024b). However, there are only a few studies examining the effect of cellular senescence on the expression and secretion of the Gal-8 protein. Wei et al. (2019) revealed that the expression of Gal-8 was robustly increased in senescent human corneal endothelial cells (HCEC) accompanied by senescence markers, such as SA-β-gal and p21. These investigators also revealed that the Gal-8 protein together with matrix metalloproteinase-2 (MMP-2) enhanced the role of senescent HCECs in corneal inflammatory neovascularization (Yu et al. 2019). It seems that in most occasions, the Gal-8 protein as well as the LILRB4 receptor evoked immunosuppression thus promoting cancer growth while alleviating the severity of inflammatory diseases.

### NKG2A/HLA-E

Pereira et al. (2019) demonstrated that the cellular senescence induced by replicative senescence, ionising radiation (X-ray), and oncogenic RAS activation stimulated a robust increase in the expression of the HLA-E protein in human fibroblasts and endothelial cells. They also reported that SASP factors, such as cytokines IL-6 and IL-8 as well as chemokine CCL2, induced the expression of HLA-E in human non-senescent fibroblasts. It is known that in human Tera-2 cells, the transcription of the *HLA-E* gene can be stimulated by IFN-γ signaling via the STAT1 pathway (Gobin and van den Elsen 2000). Pereira et al. (2019) reported that the HLA-E protein targeted the NKG2A receptor on the surface of NK and CD8^+^ T cells. Subsequently, they revealed that an inhibition of the expression of HLA-E in senescent human fibroblasts enhanced their immune elimination in vitro by NK and CD8^+^ T cells. Moreover, Pereira et al. (2019) performed histochemical stainings for HLA-E and p16INK4A, a marker of cellular senescence, and they observed that these proteins were co-localized in fibroblasts in the skin samples from aged humans. These results indicate that senescent cells are able to evade immune clearance by NK and CD8^+^ T cells by stimulating the expression of the HLA-E protein. The HLA-E protein can also be packaged into EVs (Jiang et al. 2021) although currently it is not known whether senescent cells can inhibit cytotoxic cells by secreting the HLA-E protein in EVs, a signaling mechanism known to be activated in senescent cells (Salminen et al. 2020).

### TIM-3/HMGB1/Gal-9/CEACAM1

There is abundant evidence that the cellular senescence upregulates the expression of HMGB1, Gal-9, and CEACAM1 in different models. HMGB1 is an alarmin which is secreted from senescent cells and many immune cells during times of tissue stress and inflammation. The HMGB1 protein can bind to certain immunoreceptors, such as TIM-3 and Toll-like receptor 4 (TLR4). Davalos et al. (2013) demonstrated that several treatments that could induce cellular senescence were able to stimulate the secretion of the HMGB1 protein from human fibroblasts. They also observed that senescent cells actively exported nuclear HMGB1 into the extracellular space. It is known that the HMGB1 protein can stimulate inflammatory responses although it seems that it can also inhibit some specific immune responses via the TIM-3 receptor (Chiba et al. 2012; Huang et al. 2022a). As described above, in the cancer microenvironment, the Gal-9 protein inhibits the cytotoxicity of NK and CD8^+^ T cells via the activation of TIM-3 signaling. Berben et al. (2020) demonstrated that the level of the Gal-9 protein progressively increased with aging in the human blood. Moreover, the Gal-9 protein was an important marker of frailty in old women (Mitchell et al. 2023). Currently, it is not known whether cellular senescence is involved in the increase in the Gal-9 level associated with aging. Tarallo et al. (2024) demonstrated that the temozolomide-induced senescence of mouse melanoma cells robustly increased the expression and secretion of the Gal-9 protein. Interestingly, silencing of mitofusin 1, a mitochondrial fusion protein, reduced the expression of SASP factors, decreased the expression of the Gal-9 protein, promoted immune cell recruitment, and delayed the growth of melanoma. These results indicate that cellular senescence controls the expression level of the Gal-9 protein.

The CEACAM1 protein is upregulated not only in cancer cells but also in diverse types of senescent cells. For instance, Sappino et al. (2012) demonstrated that the inducers of DNA double-strand breaks, such as neocarzinostatin and etoposide, robustly stimulated the expression of CEACAM1 and senescence markers in human epithelial cells and colon cancer cells. They revealed that the upregulation of this protein was mediated through the ATM/p53 signaling pathway. Moreover, they reported that the silencing of CEACAM1 expression impaired the p53-induced cellular senescence. Lee et al. (2024) also demonstrated that two DNA damaging agents, cisplatin and doxorubicin, increased the expression of CEACAM1 and many SASP factors in human breast cancer cells. There are observations indicating that the expression of CEACAM1 promotes vascular aging in humans and mice. For instance, Kleefeldt et al. (2019) reported that the aging process increased the expression of CEACAM1 in human internal thoracic artery and murine aorta. An age-related increase in the expression of CEACAM1 augmented oxidative stress and the expression of TNF-α as well as consequently, it increased collagen accumulation and impaired the integrity of the endothelial barrier. Currently, it is not known whether an increased expression of CEACAM1 is associated with the TIM-3-induced vascular disturbances (Cong et al. 2020).

### SIRPα/CD47

Schloesser et al. (2023) demonstrated that an increase in the expression of the CD47 protein was a hallmark of cellular senescence induced by different senescence inducers in many human and mouse fibroblast populations. These results are in agreement with the reports that the expression of CD47 increased with aging in mouse tissues, e.g. in the lungs (Tabula Muris Consortium 2020; Schloesser et al. 2023). This implies that senescent cells upregulate the expression of the marker-of-self, i.e., the CD47 protein, to evade their elimination through phagocytosis. Interestingly, Schloesser et al. (2023) revealed that senescent human fibroblasts were resistant to their efferocytosis by human macrophages. Moreover, they reported that senescent fibroblasts also inhibited the capacity of macrophages to engulf surrounding cell corpses. The suppression of macrophage-induced phagocytosis was dependent on the cell–cell contact with senescent fibroblasts rather than with secreted SASP factors. They also revealed that the inhibition of phagocytic activity of macrophages was attributed to the activation of SHP-1 by means of the ITIM domain in the SIRPα protein. Kojima et al. (2016) reported that exposure to CD47 blocking antibodies restored the phagocytic activity of macrophages and subsequently inhibited the severity of atherosclerosis in mouse models. As described above, the CD47 protein is also a receptor for the TSP1 protein and this regulation mechanism is independent of SIRPα signaling. For instance, TSP1 signaling via the CD47 receptor induces cellular senescence in endothelial cells (Gao et al. 2016; Bitar 2019). Moreover, there are observations indicating that TSP1/CD47 signaling disturbs the normal function of arteries and promotes a vascular aging process. Ghimire et al. (2020) demonstrated that the expression levels of both TSP1 and CD47 significantly increased in human and mouse arteries. They observed that the expression of the CD47 protein reduced the migration of mouse endothelial cell and the formation of capillary tubes. Ghimire et al. (2020) also reported that the angiogenic activity was significantly greater in old CD47-null mice than in their aged wild-type counterparts. The blood flow in the hind limbs was also significantly greater in the CD47-null mice than in wild-type mice. However, it should be emphasized that the TSP1 protein has many other targets which could affect blood flow with aging. In fact, the CD47 protein can act as the ligand for the SIRPα receptor as well as concurrently acting as the receptor of the TSP1 protein.

## Cooperation between inhibitory immune checkpoints and immunosuppressive cells

**Fig. 2:**
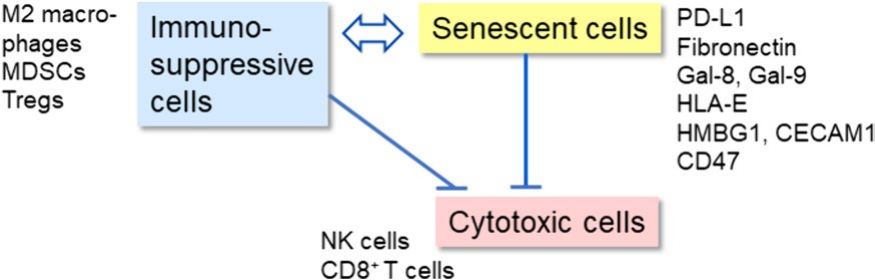
Cooperation between inhibitory immune checkpoints and immunosuppressive cells. Senescent cells suppress the functions of cytotoxic cells via inhibitory checkpoint ligands. Senescent cells also secrete chemokines which recruit immunosuppressive cells into senescent tissues. Senescent cells can also induce the differentiation of immunosuppressive cells via the PD-1/PD-L1 signaling. On the other hand, immunosuppressive cells secrete anti-inflammatory cytokines which enhance the senescence process itself and stimulate the expression of inhibitory checkpoint ligands in senescent cells. *MDSC* myeloid-derived suppressor cell, *NK* natural killer cell. Other abbreviations are as in Fig. 1

The aging process is associated with a gradual decline and remodelling of the properties of the immune system, i.e., a phenomenon called immunosenescence (Fulop et al. 2018; Salminen 2021; Santoro et al. 2021). However, immunosenescence is not only connected to the aging process since many age-related and chronic inflammatory diseases are associated with a clear immune suppressive state (Kanterman et al. 2012; Barbe-Tuana et al. 2020). Interestingly, an important hallmark of immunosenescence is the decline in the activity of cytotoxic cells, including NK and CD8^+^ T cells (Martinez-Zamudio et al. 2021; Salminen 2021; Brauning et al. 2022). An accumulation of senescent cells with enriched inhibitory immune checkpoint ligands could represent a mechanism to explain the increase in tissue immunotolerance with aging. Moreover, there is abundant evidence that aged tissues contain an increased number of immunosuppressive cells, such as MDSCs, Tregs, and M2 macrophages (Grizzle et al. 2007; Enioutina et al. 2011; Garg et al. 2014; Ruhland et al. 2016; Salminen 2020, 2022). Currently, it is known that the cooperation between inhibitory immune checkpoint proteins and immunosuppressive cells can inhibit the elimination of senescent cells by cytotoxic NK and CD8^+^ T cells (Fig. 2).

Chemokines are well characterized SASP factors secreted by pro-inflammatory senescent cells (Rodier et al. 2009; Freund et al. 2010; Acosta et al. 2013). For instance, cultured senescent cells secrete many members of two families of chemokines, i.e., CCL2,3,5,8 and CXCL1,2,3,5,10. Moreover, granulocyte–macrophage colony-stimulating factor (GM-CSF) is highly secreted by human senescent fibroblasts (Freund et al. 2010). Moreover, single-cell studies have revealed that fibroblasts isolated from aged tissues abundantly express and secrete chemokines (Salzer et al. 2018; Sole-Boldo et al. 2020). The GM-CSF and chemokines secreted from senescent cells induce haematopoiesis in the bone marrow producing myeloid cells which consequently can be recruited into inflamed tissues. For instance, GM-CSF and certain chemokines coordinate the development of immunosuppressive MDSC via myelopoiesis (Li et al. 2022). Accordingly, Hotta et al. (2019) reported that in mice GM-CSF therapy increased the expansion of Tregs. Moreover, it is known that chemokine exposure can augment the polarization of human and mouse M2 macrophages in inflammatory conditions (Mantovani et al. 2004). These studies indicated that senescent cells by secreting GM-CSF and chemokines can recruit and even stimulate the generation of immunosuppressive cells (Fig. 2).

Given that inhibitory immune checkpoints and Tregs have a crucial role in the maintenance of peripheral tolerance, it is not surprising that they are able to cooperate with each other. Francisco et al. (2009) demonstrated that PD-L1 signaling, stimulated by either PD-L1-positive immune cells or PD-L1-bound beads, was able to convert mouse naïve CD4^+^ T cells into induced Tregs (iTreg). They also reported that iTreg cells suppressed the activity of CD4^+^ T effector cells. DiDomenico et al. (2018) revealed that mouse PD-L1 ligands stimulated the expansion of iTregs by binding to the PD-1 receptor of the CD4^+^ T cells. They also reported that the PD-L1-induced Tregs inhibited the proliferation of naïve CD4^+^ T cells. Moreover, DiDomenico et al. (2018) demonstrated that the blockade of the PD-1 receptor suppressed the expansion of iTregs in a murine glioma model. It is also known that activation of the PD-1/PD-L1 checkpoint pathway stimulated the polarization of macrophages toward the immune suppressive M2 phenotype. Wei et al. (2021) reported that when human THP-1 cells were treated with human recombinant PD-L1 protein, this promoted their polarization toward the M2 macrophage phenotype. The M2 polarization was induced through the ERK/AKT/mTOR signaling pathway in human THP-1 cells. They also revealed that treatment with the PD-1 antagonist, nivolumab, inhibited the PD-L1-induced M2 polarization. There are also indications that the NF-κB-mediated increase in the expression of the PD-1 protein in MDSCs increased their proliferation in the tumor milieu (Nam et al. 2019). It is known that immunosuppressive cells are potent inhibitors of the cytotoxic NK and CD8^+^ T cells by secreting TGF-β cytokine, a mediator of interactions between immunosuppressive cells (Thomas and Massague 2005; Ralainirina et al. 2007; Flavell et al. 2010). Flavell et al. (2010) have reviewed the role of TGF-β in the suppression of cytotoxicity induced by NK and CD8^+^ T cells. It seems that the immunosuppressive microenvironment induced by MDSCs, Tregs, and M2 macrophages effectively suppresses the clearance of senescent cells from aged tissues (Fig. 2).

Immunosuppressive cells, either recruited or polarized in aging tissues, secrete anti-inflammatory cytokines, such as TGF-β and IL-10, which can promote the senescence process through two different mechanisms, (i) they can enhance the senescence process itself, or (ii) they can stimulate the expression of inhibitory checkpoint ligands in senescent cells and thus prevent their elimination. Several factors in the senescence microenvironment can activate the latent TGF-β protein, such as reactive oxygen species (ROS) and integrin β3 protein (Frippiat et al. 2001; Rapisarda et al. 2017). Lyu et al. (2018) demonstrated that the ROS-induced TGF-β signaling altered the epigenetic H4K20me3 status by increasing the expression of miR-29 which suppressed the expression of SUV4-20 h and consequently reduced the methylation status of the H4K20me site in mouse embryonic fibroblasts. The loss of H4K20me3 reduced the integrity of DNA and caused a premature fibroblast senescence. They also reported that an increase in TGF/miR-29 signaling promoted the aging process in mouse cardiac muscle. Several other studies have revealed that exposure to TGF-β increased cellular senescence in several experimental models (Tominaga and Suzuki 2019; Matsuda et al. 2023). Cellular senescence is not the only response induced by TGF-β exposure since TGF-β is a robust enhancer of age-related tissue fibrosis (Ren et al. 2023). Moreover, the IL-10 cytokine can also trigger cellular senescence via STAT3 signaling, e.g., in hepatic stellate cells (Huang et al. 2020). These examples indicate that immunosuppressive cells are potent enhancers of cellular senescence both as encountered in aging and in many age-related inflammatory diseases.

Immunosuppressive cells not only enhance cellular senescence but they are also able to stimulate the expression of many inhibitory immune checkpoint proteins, thus promoting immunosenescence in aged tissues (Fig. 2). For instance, Park et al. (2016) demonstrated that TGF-β1 treatment increased the expression of the PD-1 receptors in activated human CD4^+^ and CD8^+^ T cells. TGF-β exposure activated the SMAD3 signaling pathway which increased the transcription of the *PDCD1* gene. Park et al. (2016) also confirmed that an increase in the expression of PD-1 decreased the function of T cells. Accordingly, Shiri et al. (2024) demonstrated that the IL-10 cytokines secreted by mouse Tregs stimulated the expression of the PD-L1 protein on myeloid cells and subsequently inhibited the immunosurveillance of cancer cells by cytotoxic immune cells. Currently, a dual blockade of the TGF-β and PD-L1 proteins has been used for therapeutic purposes (Gulley et al. 2022). There are several investigations indicating that when human fibroblasts were exposed to TGF-β this stimulated the expression of fibronectin, a major ligand for the LILRB4 receptor (Varga et al. 1987; Roberts et al. 1988). On the other hand, it seems that exposure to TGF-β can inhibit the expression of activating immune checkpoint receptors. Castriconi et al. (2003) demonstrated that the treatment of human NK cells with TGF-β1 inhibited the surface expression of NKp30 and NKG2D receptors, which are major activating checkpoint receptors involved in the immunosurveillance by NK cells. They also confirmed that TGF-β1 exposure prevented the elimination of inducible dendritic cells (iDC). This suggests that TGF-β and probably other immunosuppressive mediators can promote the development of immunosuppressive states in two ways, i.e., either by activating inhibitory checkpoints or inhibiting activating checkpoints.

In conclusion, it seems that the inhibitory checkpoint receptors and their ligands of immune and non-immune cells cooperate with recruited and tissue-resident immunosuppressive cells in aged tissues and in age-related diseases. This immunosuppressive network prevents the elimination of senescent cells from aged tissues. Paradoxically, it seems that pro-inflammatory senescent cells promote tissue inflammation in aged tissues, whereas increased expression of the ligands for inhibitory checkpoint receptors in senescent cells prevents excessive devastating inflammation with aging thus maintaining the chronic low-grade inflammatory state.

## Blockade of inhibitory immune checkpoints could provide a novel senolytic therapy

Given that there are studies indicating that the clearance of senescent cells can delay the aging-associated disorders (Baker et al. 2011; Chaib et al. 2022), there has been intensive research to discover either chemical drugs or forms of immunotherapy to eliminate senescent cells from aging tissues (Sikora et al. 2019; Chaib et al. 2022; Krzystyniak et al. 2022). Immunotherapies based on the blockade of inhibitory checkpoints are in clinical use for the treatment of diverse cancers (Shiravand et al. 2022). Antibodies to the PD-1, PD-L1, and CTLA-4 proteins have demonstrated good efficacy in certain cancer types but unfortunately not in all cancers. Currently there are also some small molecule inhibitors and peptides under examination which have been claimed to block the properties of certain inhibitory checkpoint proteins (Fuchs et al. 2024). There are also some preclinical ongoing investigations examining the pros and cons of blockade of immune checkpoints in cardiovascular diseases (Suda et al. 2023), infectious diseases (Wykes and Lewin 2018), and idiopathic pulmonary fibrosis (Tan et al. 2024). Several cancer studies have compared the efficacy of the PD-1/PD-L1 blockade therapy between young and old patients. However, a meta-analysis did not find any differences in treatment efficacy between young and elderly patients (Elias et al. 2018).

The earlier experiments on the elimination of senescent cells used genetic techniques for the removal of senescent cells (Baker et al. 2011). In fact, there are rather few studies which have examined whether the blockade of the PD-1/PD-L1 axis could eliminate senescent cells in tissues, whereas many senolytic trials have utilized chemical approaches (Chaib et al. 2022). Wang et al (2022a) demonstrated that a blockade of the PD-1/PD-L1 axis enhanced immunosurveillance of senescent cells in mouse kidney, liver, and lung and moreover, it also reduced some age-related disorders. They used the anti-PD-1 therapy protocol in aged mice and thus it seems that this treatment prevented the PD-L1-induced exhaustion of CD8^+^ T cells. Currently, it is known that there are some adverse events, such as systemic inflammatory syndromes, associated with the use of immune checkpoint blockade therapies (Baldini et al. 2020; Zheng 2023). For instance, Baldini et al. (2020) reported that an anti-PD-L1 therapy for solid tumors induced immune-related adverse events more frequently in the elderly than in young patients. This could be attributed to an increase in the expression of the PD-L1 receptor in aged tissues and its wide distribution in the body. Nonetheless, there are many ongoing experiments with inhibitors of other inhibitory checkpoints. For instance, Kojima et al. (2016) demonstrated that the blocking antibodies of the CD47 protein attenuated the severity of atherosclerosis in mouse models by improving the phagocytosis capacity of immune cells and enhanced the clearance of abnormal cells from mouse vascular tissues.

## Conclusions

Given that the aging process is associated with a gradual increase in the inflammatory state, it is not surprising that as a counteracting effect there is an activation of immunosuppressive mechanisms, i.e., an increase in the expression of several inhibitory immune checkpoint proteins as well as an upregulation of immunosuppressive cells in tissues, both via recruitment into tissues and alterations in the polarization of tisssue-resident immune cells. Moreover, there are some other mechanisms to counteract increasing inflammation with aging. For instance, an increase in the aging-linked activation of NF-κB system (Salminen et al. 2008a) can be suppressed, e.g., by stimulating the expression of heat-shock proteins HSP70 and HSP90 (Salminen et al. 2008b; van Eden et al. 2017) and inducing an endotoxin tolerance state (Chan et al. 2005). Moreover, it is known that there are many metabolic immune checkpoints, such as the adenosine-induced immunosuppression (Ohta 2016). Currently, the role of cellular senescence in physiology and pathology is under debate; while it seems that it has beneficial effects during embryogenesis and development, however in some pathological conditions, such as in cancer and wound healing, it can evoke both beneficial and harmful responses in a context-dependent manner (Huang et al. 2022b). Currently, it is not known whether the upregulation of inhibitory checkpoint expression is also a context-dependent process with aging. The age-related cellular senescence seems to be a paradoxical process since senescent cells possess both pro-inflammatory and immunosuppressive properties. However, it is known that many signaling pathways which stimulate the expression of the PD-L1 ligand are well-known enhancers of the aging process, such as epigenetic regulation as well as signaling via the mTOR-related, cGAS-STING, and AhR pathways (Salminen 2024). Moreover, the anti-aging treatments with metformin and rapamycin reduce the expression levels of PD-L1 ligand protein. It does seem that inhibitory immune checkpoint signaling has an important role in the aging process.

## Data Availability

No datasets were generated or analysed during the current study.
